# Overexpression of a Transcription Factor Increases Lipid Content in a Woody Perennial *Jatropha curcas*

**DOI:** 10.3389/fpls.2018.01479

**Published:** 2018-10-22

**Authors:** Jian Ye, Chunming Wang, Yanwei Sun, Jing Qu, Huizhu Mao, Nam-Hai Chua

**Affiliations:** ^1^Temasek Life Sciences Laboratory, National University of Singapore, Singapore, Singapore; ^2^State Key Laboratory of Plant Genomics, Institute of Microbiology, Chinese Academy of Sciences, Beijing, China; ^3^State Key Laboratory of Crop Genetics and Germplasm Enhancement, Nanjing Agriculture University, Nanjing, China; ^4^Laboratory of Plant Molecular Biology, Rockefeller University, New York, NY, United States

**Keywords:** *Jatropha*, WRI1, QTL, lipid content, biofuel

## Abstract

Vegetable oil is an important renewable resource for dietary consumption for human and livestock, and more recently for biodiesel production. Lipid traits in crops are controlled by multiple quantitative trait loci (QTLs) and each of them has a small effect on lipid traits. So far, there is limited success to increase lipid yield and improve lipid quality in plants. Here, we reported the identification of a homolog of APETALA2 (AP2) transcription factor WRINKLED1 (JcWRI1) from an oleaginous plant *Jatropha curcas* and characterized its function in *Jatropha* and *Arabidopsis thaliana*. Using physical mapping data, we located *JcWRI1* in a QTL region specifying high oleate and lipid content in *Jatropha*. Overexpression of *JcWRI1* in *Jatropha* elevated seed lipid content and increased seed mass. Lipid profile in seeds of over-expression plants showed higher oleate content which will be beneficial to improve biodiesel quality. Overexpression of *JcWRI1* activated lipid-related gene expression and JcWRI1 was shown to directly bind to the AW-box of promoters of some of these genes. In conclusion, we were able to increase seed lipid content and improve seed lipid quality in *Jatropha* by manipulating one key transcription factor JcWRI1.

## Introduction

Increasing global population and emerging economies have led to the increased consumption of vegetable oil and disturbed the balance between use of edible lipids and industrial lipids (Carroll and Somerville, 2008). Considered as a promising bioenergy crop *Jatropha curcas* L. (hereinafter, *Jatropha*) offers a good solution to the “food versus fuel” debate because of its high seed lipid content, tolerance to drought and ability to grow on arid soil (Bailis and Baka, 2010; Almeida et al., 2011; Omar et al., 2011; Kumar et al., 2012; Maghuly and Laimer, 2013). Indeed, the *Jatropha* research and development communities have made tremendous progress using breeding programs to increase seed yield and also employed transgenic technologies to enhance agronomic traits in recent years, but the seed yield per *Jatropha* plant still requires much improvement for this energy crop to become competitive especially under market condition of low crude lipid price (Johnson et al., 2011).

Vegetable lipid traits including yield and quality are determined by many QTLs that are regions of DNA contributing to particular phenotypes (Zheng et al., 2008; King et al., 2015). Previous studies have reported multiple QTLs associated with lipid traits but the molecular basis of lipid QTLs remains largely unknown (Zheng et al., 2008; King et al., 2015). Major lipid biosynthetic and storage enzymes have been identified in the model plant *Arabidopsis thaliana* and other plants (Ruuska et al., 2002). Other than *Arabidopsis*, little advance has been made in the biotechnological improvements of lipid content in oleaginous crops such as rapeseed, soybean, and peanut. There are significant differences in the complexity and regulation of lipid biosynthesis between model plants and oleaginous crops (Durrett et al., 2008). For example, manipulation of a single biochemical step, such as that catalyzed by acyl-CoA:diacylglycerol acyltransferase, a bottleneck enzyme, has been proven to be of limited efficacy to upregulate the entire lipid synthesis pathway; in fact, such manipulation often leads to weak or even no impact on lipid yield (Burgal et al., 2008).

Instead of manipulation a single enzyme in lipid biosynthesis, an alternative strategy is to alter the activity of transcription factors which may regulate en bloc multiple related genes of the lipid biosynthetic pathway. It is known that during seed development transcript levels of many lipid biosynthetic genes are coordinately regulated by specific transcription factors. The *Arabidopsis* AP2 type transcription factor, WRI1, has been shown to be required for accumulation of TAG by regulating carbon flux and carbon partitioning between carbohydrate and storage lipids (Focks and Benning, 1998; Baud et al., 2007). Recently, AtWRI1 has been shown to interact with a number of proteins forming a network to regulate the transcription levels of genes in lipid biosynthetic and glycolytic pathways (Kim et al., 2016; Ma et al., 2016; Li et al., 2017; Zhai et al., 2017). Manipulation of *WRI1* or *WRI1* homologs from other plants has increased seed lipid content in *Arabidopsis* (Cernac and Benning, 2004; Liu et al., 2010; Shen et al., 2010; Pouvreau et al., 2011; Qu et al., 2012b; Grimberg et al., 2015; Jin et al., 2017). Moreover, silencing cotton *WRI1* expression resulted in increased fiber length but reduced seed lipid content suggesting the possibility of increasing fiber length *via* carbon flow repartition (Qu et al., 2012b). However, despite considerable advances, our knowledge of factors controlling lipid accumulation in oleaginous crops remains poor.

During the last decade, research on *J. curcas* has been centered on optimizing agriculture traits, particularly those controlling flowering time, disease resistance, and free FA levels in seeds. Using reverse genetic strategy, we have identified several *J. curcas* genes important for lipid quality and yield (Ye et al., 2009; Qu et al., 2012a). By genetic modification of genes encoding key enzymes in lipid biosynthesis and degradation pathways, we have successfully improved lipid quality and yield for *J. curcas* (Qu et al., 2012a). However, there has been limited work on global transcription regulation of lipid metabolic pathway in *Jatropha* seed. Using 506 microsatellite and SNP markers, we have established a first-generation microsatellite-based genetic linkage map of *Jatropha*, covering 11 linkage groups (Liu P. et al., 2011). By means of forward genetics and candidate gene mapping methods, we have successfully identified a gene encoding an AP2 transcription factor WRI1 (JcWRI1) that is located in a high-oleate QTL affecting *Jatropha* seed lipid and oleate contents. Over-expression from a *CaMV 35S* promoter results in a heritable increase of lipid content, with much higher seed lipid content and seeds mass. Lipid profile in *JcWRI1* overexpression lines is also changed, with much higher oleate content and much lower linoleate level in seeds.

## Materials and Methods

### *J. curcas* Materials and Transformation Protocol

Seeds were obtained from the *J. curcas* (*Jc*-MD 16, wild type, WT) elite plants which were pre-selected by Drs. Yan Hong and Chenxin Yi (JOil). Plants were grown in a greenhouse under natural photoperiods and ambient temperature (ranged from 25 to 35°C) in Singapore.

Transformation protocol had been previously described, mainly including co-cultivation, shoot regeneration, shoot elongation, and rooting (Qu et al., 2012a; Ye et al., 2014a).

For co-cultivation, we used small cotyledons pieces (5 mm × 5 mm) as explants which were immersed in the *Agrobacterium* cell suspension in medium II for 10–20 min. Explants were then incubated on the solid co-cultivation medium for 2–3 days at 22°C in the dark. Next, explants were transferred to callus formation medium in the dark at 25°C for 3 weeks after rinsing with sterile water till to clarification and soaked in 300 mg/L cefotaxime for 5–10 min.

For shoot regeneration, we transferred explants with newly emerged antibiotic resistant callus to shoot regeneration medium I for 3 weeks at 25°C under 16-h light/8-h dark cycles. Subsequently, any shoots regenerated from callus were transferred to shoot regeneration medium II and any callus with no regenerated shoots were transferred to shoot regeneration medium III for further regeneration.

For shoot elongation, we transferred the regenerated shoots to shoot elongation medium for elongation after 4 weeks.

For rooting, elongated shoots of about 2.5 cm were rooted on a rooting medium.

Inter-species crosses between *J. curcas* MD16 and *Jatropha integerrima* S001 was used to generate hybrid CI7041 (Ye et al., 2014b). A BC1F1 population was generated consisting of 296 individuals derived from the backcross MD16 X CI7041. The population and parental lines were planted under standard growth conditions in an experimental field located in Lim Chu Kang, Singapore. Association and LD mapping was conducted with the polymorphic markers we have developed in *J. curcas* and *J. integerrima* (Supplementary Table S1).

### Transgenic Plasmid Construction and Materials

pCAMBIA1300-derived vectors were used for all vector construction. *JcWRI1* or *JiWRI1* full length cDNA was PCR amplified using *J. curcas* and *J. integerrima* cDNA as template, respectively, with forward primer 5′-AATCGGATCCTAATGAAGAGGTCTTCTGCT-3′ and reverse primer 5′-TCATGTTAATTAATCAAACAGAATAGTTACAAGAAA-3′. After BamHI and PacI restriction enzyme digestion, the PCR products were inserted into a vector carrying a *CaMV 35S* promoter or an *Oleosin* promoter derived from oil palm (Jin et al., 2017). These vectors were used for *Jatropha* or *Arabidopsis* transformation. An ATA insertion *JcWRI1* mutant was generated by PCR amplification with primer ATGAAGAGGTCTTCTGCTTCATCTTGCTCTTCTTATACTTCTTCTTCTTCTTCTTCT according to the instruction of the Quick-Change II site-directed mutagenesis kit (Stratagene). *Arabidopsis* transformations were performed using the floral dip method (Clough and Bent, 1998).

### Lipid Analysis

Lipid analysis was performed using reagents and methods previously described (Ye et al., 2009; Qu et al., 2012a). *Jatropha* seed endosperm was carefully separated from the embryo. The dry endosperm part was ground to a fine powder and lipids were extracted with hexane three times. The combined supernatant was transferred to a glass vial and hexane was evaporated with a flow of dry nitrogen gas at 50°C. The weight of the total lipid was determined and the total lipid content was recorded as the percentage of total lipid weight to the weight of the dried endosperm (lipid weight/endosperm weight). For WT plants, the data were presented as means ± standard deviations with at least three independent biological replicates. For transgenic plants, the result were derived from at least two lines and were presented as means ± standard deviations with at least three independent biological replicates for each line.

About 10 mg of lipid was transmethylated with 3 N methanolic HCl (Sigma, St. Louis, MO, United States) plus 400 μL 2,2, dimethoxypropane (Sigma). The resultant FAMEs were separated by GC and detected/analyzed using Shimadzu GC-MS QP2010 ULTRA. GC analysis was performed at 140°C for 50 s and 30°C per minute ramp to 240°C, and the final temperature was maintained for 50 s. Peaks were identified based on their retention times compared with a FAME reference mixture (Sigma). The value of lipid composition was calculated based on the peak area as a percentage of total lipid. For WT plants, the data were presented as means ± standard deviations with at least three independent biological replicates. For transgenic plants, the result were derived from at least two lines and were presented as means ± standard deviations with at least three independent biological replicates for each line.

Total lipid was extracted and transmethylated from 100 dry *Arabidopsis* seeds as described (Jin et al., 2017). FA methyl esters were generated and separated by GC and detected using Shimadzu GC-MS QP2010 ULTRA. For WT plants, the data were presented as means ± standard deviations with at least three independent biological replicates. For transgenic plants, the result were derived from at least two lines and were presented as means ± standard deviations with at least three independent biological replicates for each line.

### RNA Extraction and Analysis

Samples of 100 mg each were ground to a fine powder in liquid nitrogen and extracted with Qiagen RNeasy Plant mini kits (Qiagen) as described. Moloney Murine Leukemia Virus Reverse Transcriptase (Promega, Madison, WI, United States) was used for RT reactions. Real-time PCR was performed with Power SYBR^®^ Green Realtime PCR Master mixture (TOYOBO) and run in *Bio-Rad* CFX96. The *J. curcas Ubiquitin* transcript level served as an internal control for seed RNA samples. *Arabidopsis Actin2* was used as an internal mRNA control. For transgenic plants, the result were derived from at least two lines. Threshold cycle values were based on three biological replicates for each line, with three technical replicates for each biological sample. Standard deviation was calculated based on the three biological replicates. See Supplementary Table S2 for PCR primer sequences.

### Confocal Experiments

Three-week-old *Nicotiana benthamiana* leaves were infiltrated with Agrobacterium as previously described. Two days after incubation, fluorescence was analyzed by confocal microscopy. The confocal laser scanning microscope technique we used was referred to the Leica SP8 microscope instruction. The images were observed from at least three biological replicates.

### Scanning Electronic Microscopy (SEM) and Light Microscopy

For observation of *Arabidopsis* seeds with the SEM, collected seeds from WT (Col-0), mutant (*wri1-4*), complemented mutant (*OLE:JcWRI1/wri1-4*), and non-complemented mutant (*OLE:JiWRI1*/*wri1-4*), respectively, were fixed with a tape inside a sample chamber, following freezing in liquid N2. Images were collected using a SEM (JSM-6360LV, JEOL, United States) with an acceleration voltage of 20 kV. For transgenic plants, the images were observed from at least two lines with 10 biological replicates for each line.

### Southern Blot Analysis

Total genomic DNA was isolated from leaves of greenhouse-grown transgenic or control plants by the cetyltrimethylammonium bromide method. Genomic DNA was digested with restriction enzymes and separated on 0.8% agarose gels. Gels were processed and transferred to a nylon Hybond-N^+^ membrane (GE Biosciences, Buckinghamshire, United Kingdom) following standard procedures. Membranes were hybridized with *Hin*dIII-treated DNA encoding the *HPT* open reading frame. The probe was labelled with [α-32P]-deoxycytidine triphosphate by random prime synthesis using Amersham Rediprime II Random Primer Labelling System (GE Biosciences). Hybridization was performed overnight at 42°C using the ULTRAHyb-Oligo hybridization buffer (Ambion, Austin, TX, United States) and signals were detected by autoradiography (Qu et al., 2012b; Ye et al., 2014a). For transgenic plants, the result were derived from at least two lines.

### ChIP Assay

About 2 g developing seeds of *J. curcas* 5 weeks after fertilization and young true leaves were used for ChIP assay. Chromatin preparation and immunoprecipitation were performed as described (Ye et al., 2015). ChIP was performed by adding WRI1 antibody and protein-A agrose beads (Kim et al., 2016). After washing, immune complexes were eluted from the protein-A beads and reverse cross-linked by incubation for at least 6 h at 65°C. Samples were treated with proteinase K for 1 h at 65°C. DNA was extracted in a final volume of 80 μL using the QIAquick PCR purification kit (Qiagen). ChIP assay was repeated with at least two transgenic lines, with two to five biological replicates for each transgenic line. One microliter of DNA was used for each real-time quantitative PCR with Bio-Rad CFX96 as described above. We used *ACTIN2* as internal control. Primers used for ChIP assays are listed in Supplementary Table S2.

### Plant Growth Condition, Agronomic Trait Collection, and Statistical Analysis

All transgenic or control plants were grown in a biosafety level 2 greenhouse according to standard practices, as described before (Qu et al., 2012a). Fruit and seed of these plants was collected and counted and their weight was taken for the whole experiment. Dry T1, T2, and T3 *Jatropha* seeds were weighed and endosperms were further analyzed for lipid content and profile. For transgenic plants, Single seed weight were measured from at least two lines with 30 biological replicates for each line.

## Results

### A Candidate Gene Associated With High Oleate Level in *Jatropha* Seeds

**FIGURE 1:**
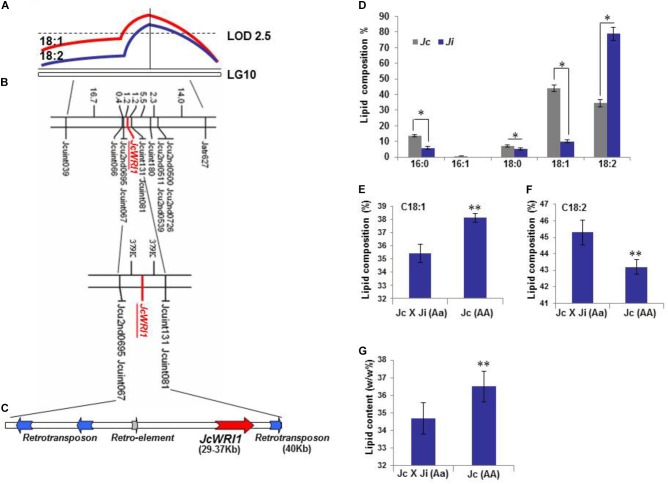
*JcWRI1* is located in a QTL underlying high-oleate seed lipid on LG10 in *Jatropha*. **(A)** A high oleate level QTL (*qHO10*) with LOD 4 on LG10 was detected in the region harboring *JcWRI1*. Horizontal line indicates 5% LOD significance threshold (2.5) based on permutation. The QTL has a strong effect on seed oleate (C18:1) levels and negative effect on seed linoleate (C18:2) acid levels. **(B)** Fine mapping of the *JcWRI1* locus. **(C)** A 40 kb contig harboring *JcWRI1* in *qHO10*. This contig is rich in transposable element. **(D)** Lipid composition of mature seeds of *J. integerrima* and *J. curcas.* Values are mean ± SD (*n* = 3) with statistical analysis using Student’s *t*-test (^∗^ indicates *p* < 0.05). **(E)** Single point analysis shows a higher seed oleate (C18:1) level in the Jc genotype (AA) than in the JcXJi (Aa) genotype. Values are mean ± SD (*n* = 3) with statistical analysis using Student’s *t*-test (^∗∗^ indicates *p* < 0.01). **(F)** Single point analysis shows a lower seed linoleate (C18:2) level in the Jc genotype (AA) than in the JcXJi (Aa) genotype. Values are mean ± SD (*n* = 3) with statistical analysis using Student’s *t*-test (^∗∗^ indicates *p* < 0.01). **(G)** Single point analysis shows higher seed lipid content in the Jc genotype (AA) than in the JcXJi (Aa) genotype. The total lipid content was recorded as the weight percentage of total lipid to dried seed (lipid weight/dried seed weight). Values are mean ± SD (*n* = 3) with statistical analysis using Student’s *t*-test (^∗∗^ indicates *p* < 0.01).

**FIGURE 2:**
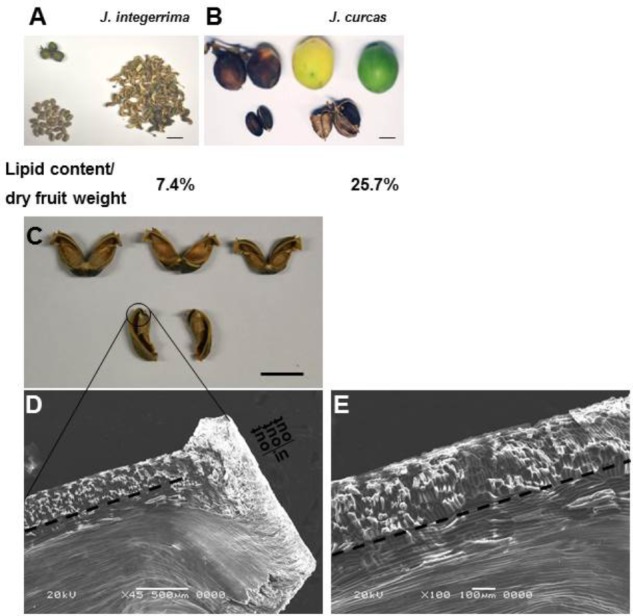
Comparisons of seeds and fruits size, lipid content in dry fruit weight of *J. integerrima* and *J. curcas*. **(A,B)** Fruits size, seeds size, fruits shell, and dry fruit lipid content of *J. integerrima* (*Ji*) and *J. curcas* (*Jc*), respectively. Noted that fiber-rich husk is the major constituents in fruits of *J. integerrima.* Values are mean ± SD (*n* = 3, total lipid weight against dry fruit weight). Bar: 20 mm. **(C)** Twisted *J. integerrima* fruit shell after drying. Bar: 10 mm. **(D,E)** Different magnification of SEM observation of twisted *J. integerrima* fruit shell. Bar of Figures 2D,E was 500 and 100 μm, respectively.

The composition of major lipid was influenced by environmental factors but it only varied slightly among cultivars of *J. curcas* (Qu et al., 2012a). By contrast, there was a significant difference in seed lipid between *J. curcas* and *J. integerrima*, a kind of plant which was affiliated to the same family as *J. curcas*. Compared with *J. curcas, J. integerrima* seeds accumulated much higher linoleate (C18:2) but lower oleate (C18:1) and also saturated FA which was not suitable to be biodiesel precursor (Figure 1D). Moreover, seeds of *J. integerrima* only accounted for 30% (wt/wt) of the whole dry fruit whereas lipid content was 7.4% based on dry fruit weight. By contrast, seeds of the high lipid yield plant *J. curcas* accounted for more than 70% of the whole dry fruit weight and lipid content was 25.7% based on dry fruit weight (Figures 2A,B). But *J. integerrima* had a fruit shell rich in carbohydrate fibers whose walls were made of layered cellulose fibrils with a regular orientation (Figures 2C–E). This structure facilitated seed dispersal during fruit drying by building up elastic energy for *J. integerrima* seeds but not for *J. curcas* seeds (Armon et al., 2011). Therefore, these results suggested that *J. integerrima* could be defective in the conversion of sucrose into precursors of lipid biosynthesis.

To further map QTL controlling lipid traits of *J. curcas*, an interspecies backcross population between *J. curcas* and *J. integerrima* was generated and a major QTL controlling two associated traits, C18:1 content and C18:2 content, was identified. This clustering of the two traits was reasonably at the same locus as the two FAs had a precursor-product relationship (Figure 1A; Liu P. et al., 2011; Wang et al., 2011; Qu et al., 2012a). The QTL s*qHO10* and *qLOL10* controlling C18:1 or C18:2 individually, account for 5.9% of variation of C18:1/C18:2 composition. The QTL controlling two associated traits was located between markers Jcuint039 and Jatr627 (Figure 1B). We had identified several key genes related to lipid biosynthesis in previous work: *JcFAD2-1* encoded a delta-12 FA desaturase, whereas *DGAT1* and *DGAT2* encoded putative acyl-CoA:diacylglycerol acyltransferases (Liu P. et al., 2011; Qu et al., 2012a). We developed SNP or SSR markers for mapping of *JcFAD2-1*, *JcDGAT1*, *JcDAGT2*, and also *JcWRI1* which encoded WRI1, an AP2 transcription factor in *Jatropha*. As only *JcWR11* was localized in the QTL region on LG10, we therefore focused on the QTL interval on LG10 harboring *JcWRI1*. We generated additional 10 markers in the QTL region (Supplementary Table S1). A final set of 12 markers covering the QTL region was identified. Meanwhile, an SSR marker (Supplementary Table S1) was directly identified in *JcWRI1*, which was finally mapped within 1.2 cM to the flanking markers of Jcu2nd0695, Jcuint067, Jcuint131, and Jcuint081 (Figure 1B). The physical distance of 1.2 cM was 580 kb based on the map length 663.0 cM and the entire genome of *J. curcas* 320.5 Mb (Liu H. et al., 2011). A high-quality assembly of a *JcWRI1*-containing 40 kb contig based on public databases as well as our own database showed no other coding gene in this region besides a few transposable elements (TEs) (Armon et al., 2011; Figure 1C).

### Effect of *JcWRI1* on Lipid Traits

Single point analysis showed that genotypes of *JcWRI1* gene and flanking markers were significantly correlated with C18:1 and C18:2 compositions (Table 1). Composite interval mapping indicated that QTLs controlling C18:1 and C18:2 compositions were localized in the region of Jcuint131-JcWRI1-Jcuint067, where *JcWRI1* was located at 1.2 cM to the flanking two markers (Figure 1B; Table 2). The additive value of C18:1 (4.75) was opposite to that of C18:2 (-4.4), consistent with the fact that C18:2 was derived from C18:1 by desaturation. Comparison of C18:1 and C18:2 content between the two *JcWRI1* genotypes showed significant effects of *JcWRI1* on the two compositions (Figures 1E,F). Further analysis of the effects of *JcWRI1* on lipid content in the QTL mapping population by using Student’s *t*-test with Bonferroni correction covered significant difference between *JcWRI1* genotypes (Figure 1G). Our results indicated that *JcWRI1* could affect not only C18:1 and C18:2 compositions but also lipid content.

**Table 1:** Single point analysis for the effect of *WR11* on lipid profile in *Jatropha* backcrossed population.

Trait	Chrom.	Marker no.	Marker	Position (cM)	*b*1	*F*(1,*n*-2)	pr(*F*)	
C18:1	10	4	Jcuint131	20.6	3.307	4.868	0.028	∗
C18:1	10	5	JcWR11	21.8	3.58	5.664	0.018	∗
C18:1	10	6	Jcuint067	23	3.635	5.887	0.016	∗
C18:2	10	4	Jcuint131	20.6	-2.973	3.482	0.063	
C18:2	10	5	JcWR11	21.8	-3.215	4.035	0.045	∗
C18:2	10	6	Jcuint067	23	-3.171	3.951	0.048	∗

**Table 2:** Composite interval mapping for *JcWRI1*.

Trait	Chromosome	Marker interval	Position(cM)	LOD	*R*2(%)	*a*
C18:1	10	Jcuint131-JcWRI1-Jcuint067	15.2	4	5.9	4.75
C18:2	10	Jcuint131-JcWRI1-Jcuint067	15.2	3	4.6	-4.4

### A *WRI1* Variant From *J. integerrima*

**FIGURE 3:**
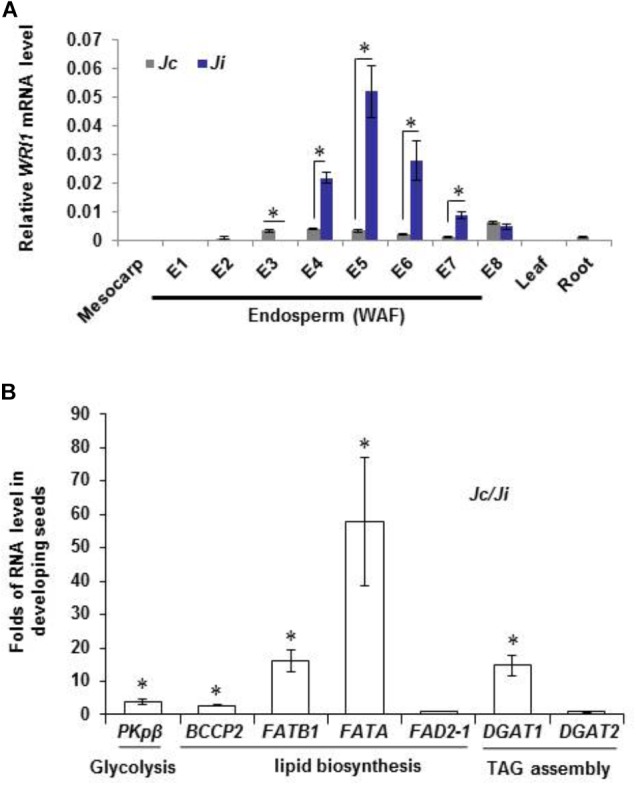
Transcripts level of *JcWRI1* and some key lipid-related genes in *J. curcas* and *J. integerrima*. **(A)** Expression levels of *WRI1* in fruit mesocarp, leaf, root, and different endosperm developmental stages (from Endosperm 1 to Endosperm 8, E1–E8) of *J. curcas* (*Jc*) and *J. integerrima* (*Ji*). E1–E8 represented samples of endosperm at different developmental stages (1–8 weeks after fertilization, WAF). The *Jatropha Ubiquitin* transcript served as an internal control for all RNA samples. Values are mean ± SD (*n* = 3) with statistical analysis by Student’s *t*-test (^∗^ indicates *p* < 0.05). **(B)** Comparison of expression levels of some key genes involved in glycolysis, lipid biosynthesis and TAG assembly in developing endosperms of *J. curcas* (*Jc*) and *J. integerrima* (*Ji*) at 6 WAF. The *Jatropha Ubiquitin* transcript served as an internal control for all RNA samples. The value was presented as fold of *Jc* to *Ji*. Values are mean ± SD (*n* = 3) with statistical analysis by Student’s *t*-test (^∗^ indicates *p* < 0.05).

We had identified *WRI1* genes from *J. curcas* (Mao et al., 2011). Figure 3A showed that the *WRI1* expression level in *J. integerrima* seeds was even higher than that in *J. curcas* seeds during the peak of lipid production (4-6 weeks after fertilization, WAF), suggesting that *WRI1* transcription level was not affected in *J. integerrima* seeds. Meanwhile, we found that some key lipid-related genes, for example, the plastidic pyruvate kinase gene *PKpβ*, biotin carboxyl carrier protein 2 gene *BCCP2*, FA thioesterase genes *FATB1* and *FATA*, and acyl-CoA:diacylglycerol acyltransferase 1 *DGAT1* were expressed at a much lower level in seeds of *J. integerrima*, compared with those of *J. curcas* (Figure 3B). This difference in expression levels may explain the low content of saturated FAs and C18:1 in *J. integerrima*. As a consequence of a lower expression of FA biosynthesis gene, such as *FATB1*, we expected the FA profile to be also changed accordingly in *J. integerrima*. Indeed, gas chromatographic analysis showed that the relative amount of the end product of FA desaturation, linoleate (C18:2), was strongly elevated, whereas the relative amount of the precursor oleate (C18:1) was most obviously decreased in seeds of *J. integerrima*. Furthermore, palmitic acid (C16:0) was also reduced to only 5.9% of the total lipid. This reduction can be explained by the lower expression of *FATB1*, which had been shown to be linked with palmitic acid (C16:0) level (Figure 1D; Ye et al., 2009).

**FIGURE 4:**
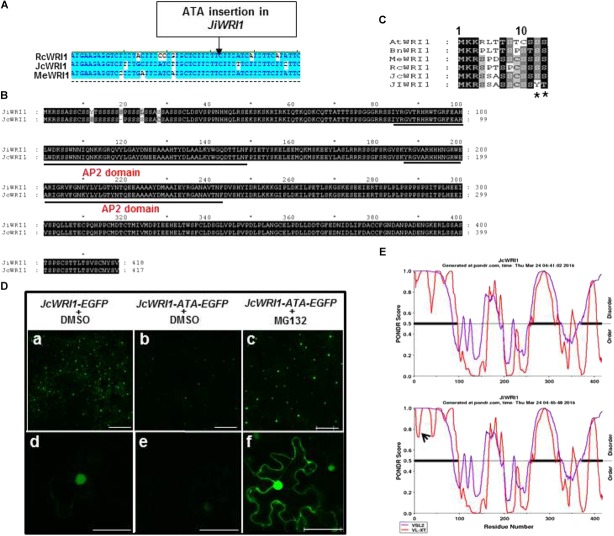
An ATA insertion in the conserved region of *JiWRI1* reduces JiWRI1 protein accumulation. **(A)** Alignment of *JcWRI1* nucleotide sequences with its Euphorbiaceae homologs: *Jatropha curcas* (*Jc*), *Ricinus communis* (*Rc*), and *Manihot esculenta* (*Me*). The ATA-insertion site for WRI1 of *J. integerrima* was indicated. **(B)** Alignment of complete JcWRI1 and JiWRI1 amino acid sequences. Underlined regions were DNA binding AP2 domains. **(C)** Alignment of N-terminal amino acid sequence (1–13aa) of JcWRI1, JiWRI1 and other WRI1 homologs. *Arabidopsis thaliana* (*At*), *Brassica napus* (*Bn*), *Manihot esculenta* (*Me*), *Ricinus communis* (*Rc*), *Jatropha curcas* (*Jc*), and *J. integerrima* (*Ji*). ^∗^ Indicates the mutated amino acid caused by ATA insertion. **(D)** Confocal images to show protein accumulation of JcWRI1, ATA-insertion mutant of JcWRI1 proteins and the latter treated with MG132. Figures of upper panel **(a–c)** were magnified in lower panel **(d–f)**. Bars of upper panel **(a–c)** and lower panel **(d–f)** are 250 and 50 μm, respectively. **(E)** Protein stability predication and disorder predication for JcWRI1 and JiWRI1 protein using two software (VSL2 and VL-XT) (https://omictools.com/vsl2-1-tool). Arrow indicates the protein disorder caused by the ATA insertion.

Further sequencing analysis of *WRI1* gene sequences from related plants showed that the *JiWRI1* gene contained a 3-nt (ATA) off-frame insertion in a highly conserved region of TCT repeat near the start codon (Figure 4A). This insertion resulted in mutation in two conserved amino acids (S12Y and S13T) and one additional serine insertion (Figures 4B,C).

To assess the significance of this ATA insertion, we fused *JcWRI1* and ATA insertion *JcWRI1* genes with *EGFP* and performed transient expression of fusion proteins in *N. benthamiana* leaves. There was much less WRI1–ATA–EGFP fusion protein accumulation in tobacco cells compared to the WT WIR1–EGFP fusion protein (Figure 4D). Upon treatment with MG132, however, WRI1–ATA–EGFP fusion protein accumulation was enhanced in both the nucleus and the cytoplasm (Figure 4D), indicating the ATA insertion may destabilize the WRI1 protein. This hypothesis was supported, in part, by bioinformatics predication (Figure 4E).

### *JcWRI1* but Not *JiWRI1* Complemented the *Arabidopsis wri1* Mutant

**FIGURE 5:**
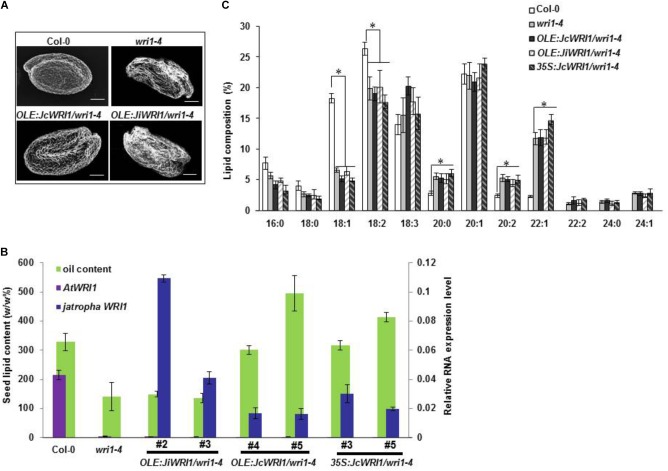
*JcWRI1* but not *JiWRI1* complements the *Arabidopsis thaliana wri1-4* mutant. **(A)** Images of mature seeds of the wild-type (Columbia-0 [Col-0] ecotype), mutant (*wri1-4*), complemented mutant (*OLE:JcWRI1/wri1-4*), and non-complemented mutant (*OLE:JiWRI1*/*wri1-4*). Size bar: 100 μm. **(B)** Seed lipid contents and *WRI1* gene expression levels of Col-0, *wri1-4*, 2 independent *wri1-4* lines complemented with *JiWRI1* transcribed from the seed specific oil palm (*Elaeis guineensis*) *Oleosin* promoter (*OLE:JiWRI1/wri1-4*), and four independent *wri1-4* lines complemented with *JcWRI1* transcribed from either the seed specific *Oleosin* promoter (*OLE:JcWRI1/wri1-4*) or a *CaMV 35S* promoter. For each transgenic line, five plants were grown. Twenty seeds were harvested from each plant and pooled. Error bars correspond to the SD calculated from three biological replicates per pool of 100 seeds. Expression levels of both *AtWRI1* and *Jatropha WRI1* (*JcWRI1* or *JiWRI1*) in complementary lines were measured by qRT-PCR using RNAs isolated from mature seeds. The *A. thaliana actin2* and the *Jatropha Ubiquitin* transcripts served as an internal control for all RNA samples. Error bars correspond to the SD calculated from seeds samples of three plants from each line. **(C)** Lipid composition in *Arabidopsis* seeds of Col-0, *wri1-4*, two independent *wri1-4* lines complemented with *JiWRI1* transcribed from the seed specific oil palm (*Elaeis guineensis*) *Oleosin* promoter (*OLE:JiWRI1/wri1-4*), and four independent *wri1-4* lines complemented with *JcWRI1* transcribed from either the seed specific *Oleosin* promoter (*OLE:JcWRI1/wri1-4*) or a *CaMV 35S* promoter. Error bars correspond to the SD calculated from three biological replicates per pool of 100 seeds. Values are mean ± SD (*n* = 3) with statistical analysis by Student’s *t*-test (^∗^ indicates *p* < 0.05).

To further investigate the functionality of *JcWRI1* and *JiWRI1* in plants, we used these two genes to complement the *wri1* mutation in *Arabidopsis*. Homozygous *Arabidopsis wri1-4* mutant plants (Baud et al., 2007) were transformed with *JcWRI1* or *JiWRI1* cDNA, expressed from a seed-specific oil palm (*Elaeis guineenis* Jacq.) *EgOleosin* promoter. For each construct, five independent transgenic lines were selected and propagated and T3 progeny plants were subjected to detailed analyses. Microscopic observation of mature dry seeds showed a complete reversion of the *wri1-4* mutant seed phenotype in the *JcWRI1* complemented lines (Figure 5A). Seed lipid content analyses confirmed that *JcWRI1* but not *JiWRI1* was able to restore defects in lipid accumulation previously found in *wri1-4* seeds. This restoration was even seen in *JcWRI1* lines with a lower transgene expression level compared to the *JiWRI1* lines (Figure 5B). Lipid profile analysis showed that neither JcWRI1 nor JiWRI1 was able to complement the lipid composition of *wri1-4* mutant (Figure 5C).

### Overexpression of *JcWRI1* Increased Seed Mass and Seed Lipid Content in *Jatropha*

**FIGURE 6:**
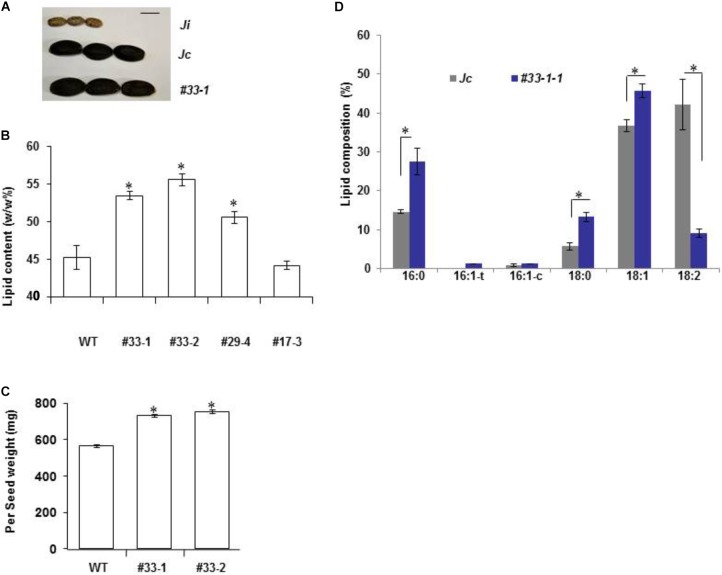
Overexpression of *JcWRI1* increases seed size and lipid content in *J. curcas* seeds. **(A)** Seed size of *J. curcas* (*Jc*), *J. integerrima* (*Ji*), and T2 seeds of transgenic *J. curcas* overexpressing *JcWRI1* (#33-1). Bar: 10 mm. **(B)** Total lipid content (lipid weight/endosperm weight) consistently increased in T2 seeds of transgenic *J. curcas* overexpressing *JcWRI1* (#33-1, #33-2, and #29-4 are corresponding T2 progeny of T1 plants shown in Supplementary Figure S1B). WT (wild type) is non-transgenic *J. curcas* control seeds. Values are mean ± SD (*n* = 10) with statistical analysis by Student’s *t*-test (^∗^ indicates *p* < 0.05). **(C)** Increase of seed weight in transgenic *J. curcas* plants overexpressing *JcWRI1*. T2 seeds (#33-1and #33-2) were used. Values are mean ± SE (*n* = 30) with statistical analysis by Student’s *t*-test (^∗^ indicates *p* < 0.05). **(D)** Statistic analysis of lipid profile in T3 seeds of *J. cucas* plants overexpressing *JcWRI1*. Values are mean ± SD of five seed replicates with statistical analysis by Student’s *t*-test (^∗^ indicates *p* < 0.05).

To further increase *Jatropha* lipid yield, we placed the *JcWRI1* cDNA under the control of a constitutive *CaMV 35S* promoter for *Jatropha* transformation. We generated 20 independent transgenic lines as shown by Southern blot analysis (Supplementary Figure S1A). There was a 10–25% increase in lipid content of the seeds of a few T1 generation transgenic lines compared with WT control (Supplementary Figure S1B). On the basis of complete T-DNA transfer, single transgene copy number and increased T1 seeds lipid content, we selected two independent lines (#33 and #29) for further T2 generation seed analysis. A transgenic line (#17) with two T-DNA insertions and no effect on lipid content in T1 seeds was used as a transgenic control (Supplementary Figures S1A,B). Consistent with the increased seed size an increase in heritable lipid content was observed in seeds of #33-1, #33-2, and #29-4 (Figures 6A,B). Interestingly, the final dry seed weight was obviously increased in #33-1 and #33-2 (Figure 6C).

### High Oleate and Palmitic Acid in *JcWRI1* Ectopic *Jatropha* Seed

Since more linolenic but less oleic and palmitic acid accumulated in a *WRI1* defective plant *J. integerrima*, we deduced that a higher oleic and palmitic acid level should be seen in *JcWRI1* overexpression seeds. Gas chromatographic analysis confirmed that the relative amount of the middle product of FA, oleic (C18:1), stearic (C18:0), and palmitic (C16:0) acid were all strongly increased whereas the relative amount of the end product linoleate (C18:2) was most obviously decreased in T3 generation *JcWRI1* overexpression seeds (Figure 6D; Supplementary Figure S1C).

### A Few Lipid Biosynthesis and Storage-Related Genes Were Directly Regulated by *JcWRI1*

**FIGURE 7:**
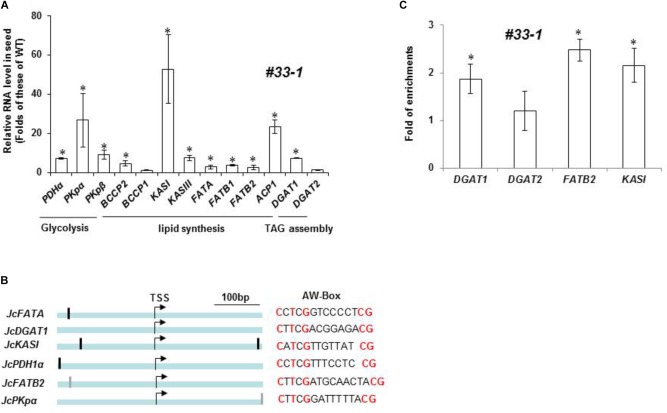
Genes involved in lipid biosynthesis and assembly pathways are differentially regulated by *JcWRI1*. **(A)** Expression levels of lipid biosynthesis pathway genes in endosperm of *J. cucas* plants overexpressing *JcWRI1* line #33-1. The *Jatropha Ubiquitin* transcript served as an internal control for all RNA samples. The value was presented as fold of the level in #33-1 against WT control (mean ± SD, *n* = 3) with statistical analysis by Student’s *t*-test (^∗^ indicates *p* < 0.05). **(B)** Diagram of gene structure of AW-box containing promoters. Dark bars in promoter regions indicate AW-box and gray bars indicate AW-like box. Bar: 100 bp. **(C)** Identification of direct target genes of JcWRI1 by Chromatin immunoprecipitation (ChIP) assay with WRI1 antibody. Developing *J. curcas* endosperm (six WAF) was harvested for ChIP assays. The fold enrichment was expressed relative to *JcTubulin2* which was used as an internal control. Amplification levels obtained with the individual primer pair served as background signal of JcWRI1 binding. The experiment was repeated twice with similar results. Values are mean ± SD of three technical replicates with statistical analysis by Student’s *t*-test (^∗^ indicates *p* < 0.05).

Since *JcWRI1* encoded a transcription factor that regulated downstream genes, we attempted to identify direct target genes of *JcWRI1*. We isolated RNAs from *Jatropha* transgenic line #33-1 seeds and performed qRT-PCR to check expression levels of *JcWRI1* and other genes involved in lipid biosynthesis and storage. The *JcWRI1* expression level was 1000-folds of WT control (Supplementary Figure S2). Several key genes in FA biosynthesis and regulation pathways were upregulated by ectopic *JcWRI1* overexpression, including genes for glycolysis, FA synthesis, and TAG assembly. Expression levels of *PDHα*, *PKpα*, *PKpβ*, *BCCP2*, *KASI*, *KASIII*, *FATA*, *FATB1*, *FATB2*, *ACP1*, and *DGAT1* were higher in #33-1 compared with WT control (Figure 7A).

**FIGURE 8:**
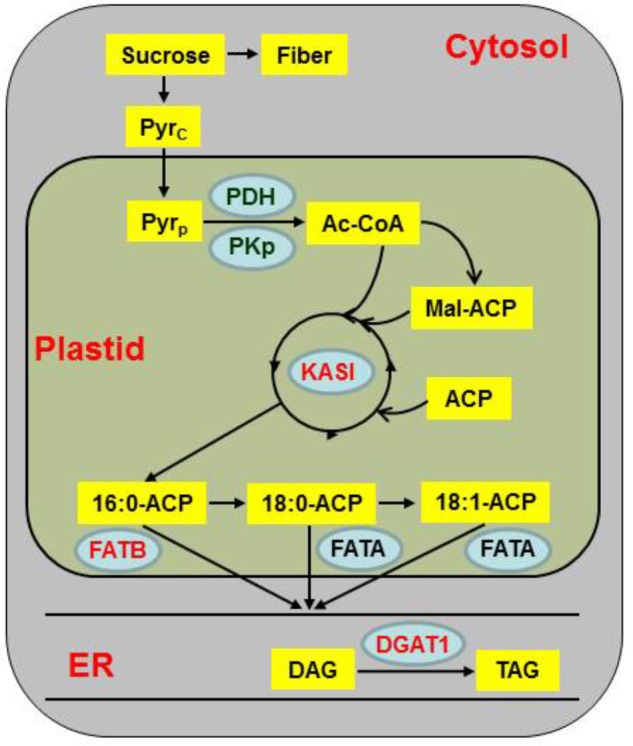
A working model for the role of JcWRI1 in both controlling carbon partitioning and lipid biosynthesis in *Jatropha* seeds. In this framework, WRI1 appears as a key gene, acting at the interplay between the carbon flow and lipid metabolism. JiWRI1 possess a loss of function due to an ATA-insertion in conserved site, resulting in a significant differences in carbon partitioning and lipid quality between the two *Jatropha* species (*Jatropha curcas* and *J. integerrima*). Overexpression of JcWRI1 in *J. curcas* increases the lipid content but also the ratio between oleate and linoleate by upregulating fatty acid biosynthesis and regulation genes. Among them, the diacylglycerol acyltransferase gene *DGAT1*, representing rate-limiting enzymes in plant lipid accumulation, the β-ketoacyl-ACP synthase I gene *KAS I*, catalyzing the initial step of FA biosynthesis and the palmitoyl ACP thioesterase gene *FATB2*, encoding enzyme for producing palmitic acids (the red fonts in the blue circles in Figure 8) were directly upregulated by JcWRI1 (Figure 7C). And the pyruvate dehydrogenase gene *PDH*, the pyruvate kinase gene *PKp*, the biotin carboxyl carrier protein 2 gene *BCCP2*, the β-ketoacyl-[acyl carrier protein] synthase III gene *KASIII*, the acyl carrier protein gene *ACP1* and the stearoyl-acyl carrier protein thioesterase gene *FATA* were indirectly upregulated by JcWRI1 (Figure 7A and the blue circles in Figure 8). The yellow boxes indicated the chemical substances participating in lipid biosynthesis. The blue circles represented the protein upregulated by JcWRI during lipid biosynthesis. Among them, the red fonts meant the corresponding genes were directly upregulated by JcWRI. Pyr, pyruvate; Ac-CoA, acetyl-CoA; ACP, acyl carrier protein; Mal-ACP, malonyl-ACP; DAG, diacylglycerol; TAG, triacylglycerol; PDH, pyruvate dehydrogenase; PKp, pyruvate kinase; KASI, β-ketoacyl-[acyl carrier protein] synthase I; FATA, stearoyl-acyl carrier protein thioesterase A; FATB, palmitoyl-acyl carrier protein thioesterase B; DGAT1, diacylglycerol acyltransferase; ER, endoplasmic reticulum.

A putative WRI1-binding motif AW-box was identified at proximal upstream regions of genes for lipid biosynthesis and assembly pathways (Figure 7B). We performed ChIP using WRI1 antibody to identify direct target genes of JcWRI1 in developing *Jatropha* seeds. Figure 7C showed that the promoters of *DGAT1*, *FATB2*, and *KASI* but not that of *DGAT2* were direct targets of JcWRI1. These genes encoded several FA synthesis enzymes (FATB2 and KASI) and an enzyme for triacylglycerol assembly (DGAT1). Our results identified the role of JcWRI1 in both controlling carbon partitioning and lipid quality in *Jatropha* seeds. Based on these results, we proposed a working model for the critical role of *Jatropha* WRI1 in carbon flow controlling and lipid biosynthesis (Figure 8). Several lipid biosynthesis and storage related genes (esp. for *KASI*, *FATB*, and *DGAT1*) were directly bound by *Jatropha* WRI1 and gene transcriptions were augmented during seed maturation. Therefore, the enhanced lipid biosynthesis consumes much more carbon hydrates and reduced the carbon flow for fiber biosynthesis in *J. curcas*. On the contrast, JiWRI1 is extremely unstably, leading to lower lipid but much higher fiber biosynthesis (Figure 2).

In contrast to its effects in *Jatropha*, ectopic expression of *JcWRI1* in *Arabidopsis* only promoted expression of genes for FA biosynthesis but not for genes related to glycolysis and TAG assembly (Supplementary Figure S3). This observation was consistent with no obvious changes in FA profile in seeds of transgenic *Arabidopsis* over-expressing JcWRI1 (Figure 5C).

## Discussion

AtWRI1 is the best understood example of transcription factors in the lipid biosynthesis regulatory network in plants. Prior to this study, overexpression of homologs of *Arabidopsis* WRI1, e.g., *Brassica napus*, *Zea mays*, and *Elaeis guineensis*, has resulted in the enhanced accumulation of *Arabidopsis* seed lipid content (Liu et al., 2010; Grimberg et al., 2015; Jin et al., 2017). Before our report, there is no oleaginous crop showing increased seed lipid content by over-expression of WRI1 homologs. Extending previous findings, we have identified WRI1 homolog from the high seed lipid plant *J. curcas* and shown that the strategy of ectopic expression can be applied to not only enhance seed lipid accumulation but also alter lipid quality in the perennial woody crop *J. curcas*.

### JcWRI1 Shows Some Atypical Characters Unlike as the Stereotypic WRI1

In *Arabidopsis*, the WRI1 transcription factor regulates transcription of glycolysis-related and FA biosynthetic genes by interacting with the MED15 subunit of the MEDIATOR complex during embryogenesis (Kim et al., 2016). As expected from the enhanced lipid traits, a subset of lipid biosynthesis genes, e.g., BCCP and KASI, was upregulated by JcWRI1 in both *Arabidopsis* and *Jatropha* (Figure 7A; Supplementary Figure S3), indicating evolutionally conserved motifs for JcWRI1 binding and activation. Interestingly, seeds of *J. integerrima*, a plant with a putative WRI1 loss-of-function mutation showed much low lipid to carbohydrate-rich fiber ratio, providing further evidence that *Jatropha* WRI1 gene is an important regulator for lipid biosynthesis.

We note that whereas *DGAT1* expression in *J. curcas* was regulated by JcWRI1, over-expression of *JcWRI1* had no effect on *AtDGAT1* in transgenic *Arabidopsis* (Figure 7A; Supplementary Figure S3). DGAT1 is a conserved enzyme for TAG assembly in plants. It is possible that the *Arabidopsis DGAT1* promoter region does not contain any AW-box which is the binding motif of JcWRI1.

The induced expression of thioesterase genes, e.g., *JcFATB* by JcWRI1 may partially explain the differential effect on FA profiles between two related *Jatropha* species. We have shown that *JcFATB* is a key factor controlling FA desaturation ratio and C16:0 FA content (Liu et al., 2010). Its lower expression in *J. integerrima* may also explain its lower C16:0 levels compared with those of *J. curcas* (Figures 1D, 3B). Furthermore, overexpression plants of WRI1, there is a higher C16:0 in *J. curcas* but no change in C16:0 levels was found in *Arabidopsis*. Not only C16:0, we observed little lipid profile changes in *Arabidopsis* seeds over-expressing *JcWRI1* (Figure 5C), which is consistent with little change in the expression of genes such as *AtFATB*.

In addition, we found the lower C18:2 but also a higher C18:1 composition in *Jatropha* seeds overexpressing *JcWRI1* (Figure 6D). We regarded the increased oleic acid (C18:1) level at the expense of linoleic acid (C18:2). Some genes, e.g., *PDHα*, *PKpα*, *PKpβ*, *BCCP2*, *KASI*, *KASIII*, *FATA*, *ACP1*, and *DGAT1* encoded enzyme mediates production of oleic acid (C18:1) are highly induced by *JcWRI1* overexpression. These genes include but not limit to *JcFATA* and *JcDGAT1*, the former encodes activity toward oleoyl-ACP and produce C18:1, which blocks the carbon flow for the synthesis of the C18:2. Furthermore, the overexpression of *DGAT1* in maize increased oil and oleic acid contents (Zheng et al., 2008).

Over-expression of *BnWRI1* resulted in the upregulation of genes involved in glycolysis, FA synthesis, lipid assembly, and flowering time in *Arabidopsis* (Liu et al., 2010). Similar to the case of transgenic *Arabidopsis* and tobacco, over-expression of *JcWRI1* also alters lipid profile in *Jatropha* seeds. We did observe the upregulation of similar classes of genes involved in glycolysis, FA synthesis, and lipid assembly in *Jatropha* plants over-expressing *JcWRI1* (Figure 7A). In contrast to *BnWRI1* over-expression there is no obvious flowering time and related gene expression changes in transgenic *Jatropha* plants over-expressing *JcWRI1* under greenhouse condition as compared with WT control. This result suggests that different WRI1 homologs from different oleaginous crops may have their own specific function for adaptation to their prevailing growth environments.

### Post-translational Stability and Function of WRI1

AtWRI1 has been reported to be unstable being regulated by post-translational modifications such as phosphorylation and ubiquitination (Zhai et al., 2017). Modifications of AtWRI1 protein through removal or changes in the PEST motif can increase AtWRI1 protein stability leading to enhanced TGA accumulation in seeds. We found that the low lipid yield *Jatropha* species, *J. integerrima* encodes a WRI1 variant with reduced protein stability. There are three intrinsically disordered regions (IDRs) in AtWRI1 and one of them was close to the N-terminus whereas the others are located in the C-terminus of the protein. AtWRI1 has been shown to be degraded *via* 26S proteasome as a result of its interaction with CULLIN3-based E3 ligase bridge BTB/POZMATH proteins. In plant cells, phosphorylation of transcription factors is well recognized as a mechanism that influences stability (Zhai et al., 2017). The serine to tyrosine mutation between JcWRI1 and JiWRI1 might be related to phosphorylation and this modification may affect the balance between FA and carbohydrate accumulation in seeds and the developmental regulation of these two related species. The phosphorylation status of WRI1 might also affect its interaction with MED15, thereby providing another possibility to explain the SNP we have identified in *Jatropha WRI1* (Kim et al., 2016).

## Conclusion

Here, we have shown that it is possible to improve lipid yield and also quality in the biodiesel plant *Jatropha* through manipulation of a key transcription factor JcWRI1.

## Author Contributions

JY and N-HC designed the experiments and drafted the manuscript. CW did QTL experiments and analyzed data. YS performed vector construction and fatty acid analysis. JQ carried out molecular analysis. HM did the *Jatropha* transformation. All authors read and approved the final manuscript.

## Conflict of Interest Statement

Patents relating to the JcWRI1 and its usage have been filed by the Temasek Life Sciences Laboratory.

## Abbreviations

ACP: acyl carrier protein
AP2: APETALA2
bp: base pairs
CaMV: *cauliflower mosaic virus*
ChIP: chromatin immunoprecipitation
DGAT: diacylglycerolacyl transferase
EGFP: enhanced green fluorescent protein
FA: fatty acid
FAD: fatty acid desaturase
FAME: fatty acid methyl ester
FATB: palmitoyl acyl-acyl carrier protein thioesterase B
GC: gas chromatography
*HPT*: *hygromycin phosphotransferase*
Jc-MD: *Jatropha curcas* MD isolate
*Jc*: *Jatropha curcas*
*Ji*: *Jatropha integerrima*
KASI: β-ketoacyl-[acyl carrier protein] synthase I
qRT-PCR: quantitative reverse transcriptase polymerase chain reaction
QTLs: quantitative trait loci
SNP: single-nucleotide polymorphism
SSR: simple sequence repeats
TAG: triacylglycerol
TE: transposable element
WRI1: WRINKLED1
