# Supplementary figures and images for: Overexpression of a Transcription Factor Increases Lipid Content in a Woody Perennial Jatropha curcas
Source: Front Plant Sci. 2018 Oct 22;9:1479. doi: 10.3389/fpls.2018.01479 (PMC6204399; doi:10.3389/fpls.2018.01479)

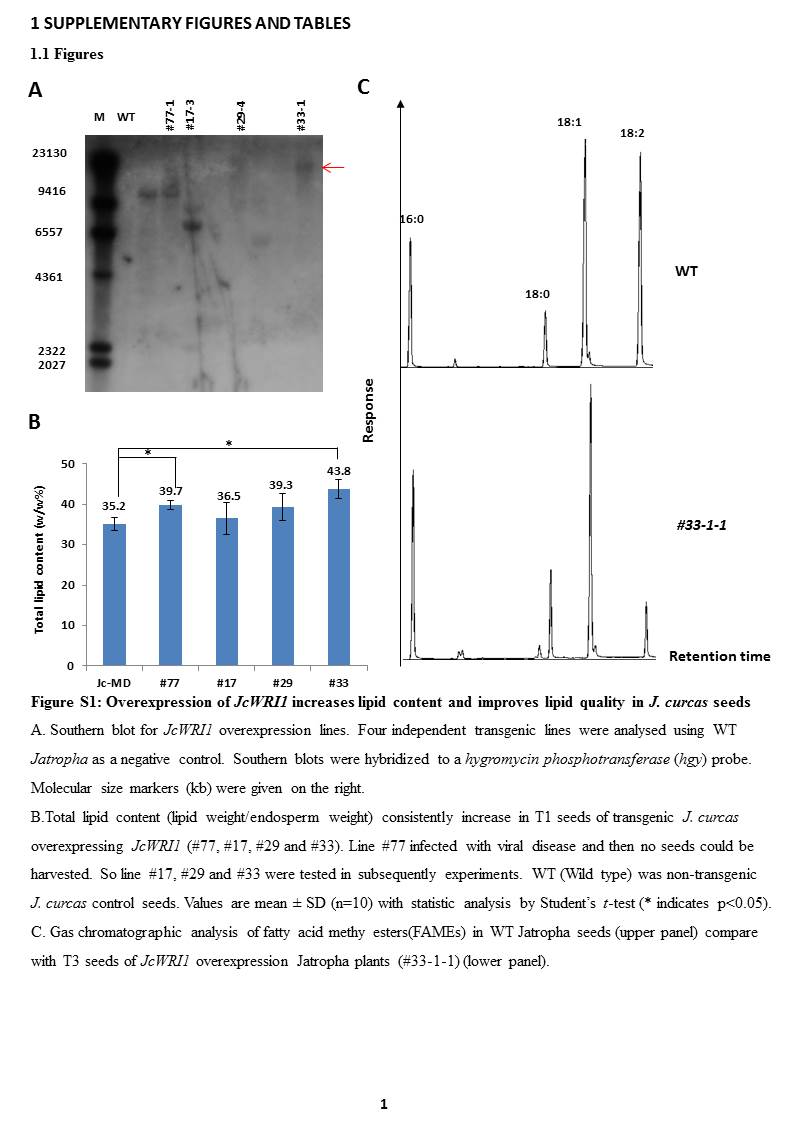

Supplement: Supplementary file 1 [file Image_1.JPEG]

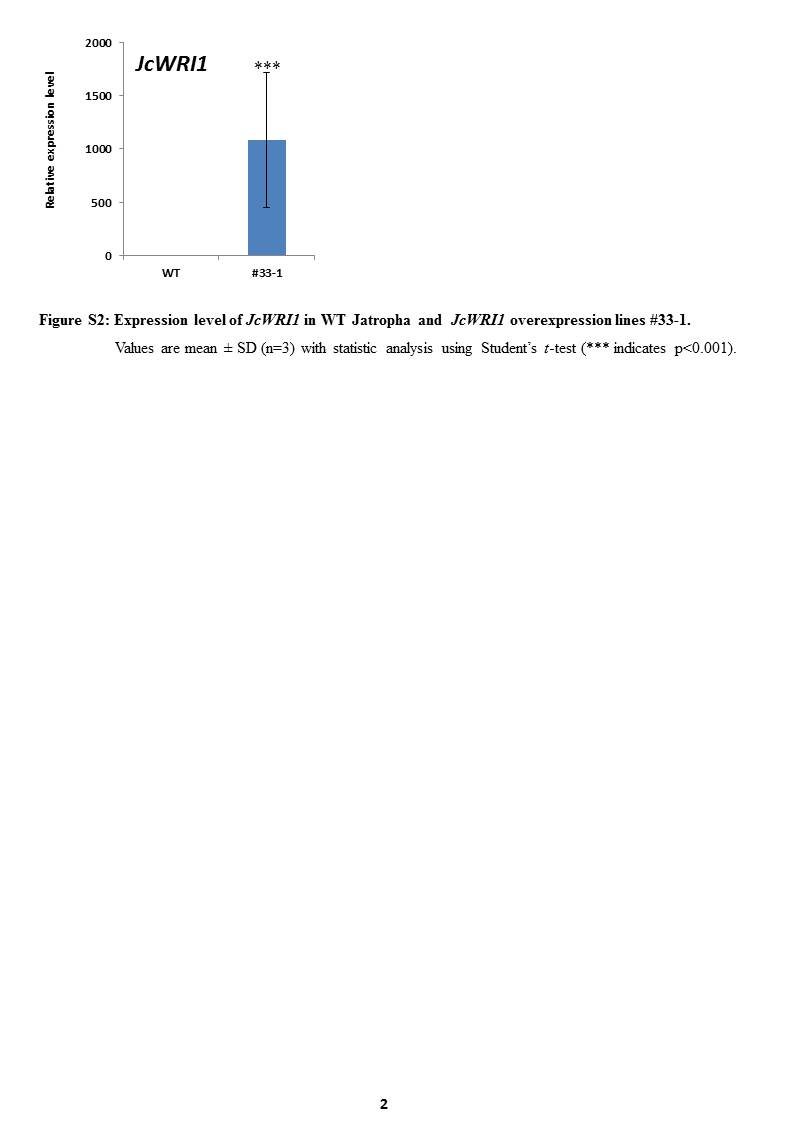

Supplement: Supplementary file 2 [file Image_2.JPEG]

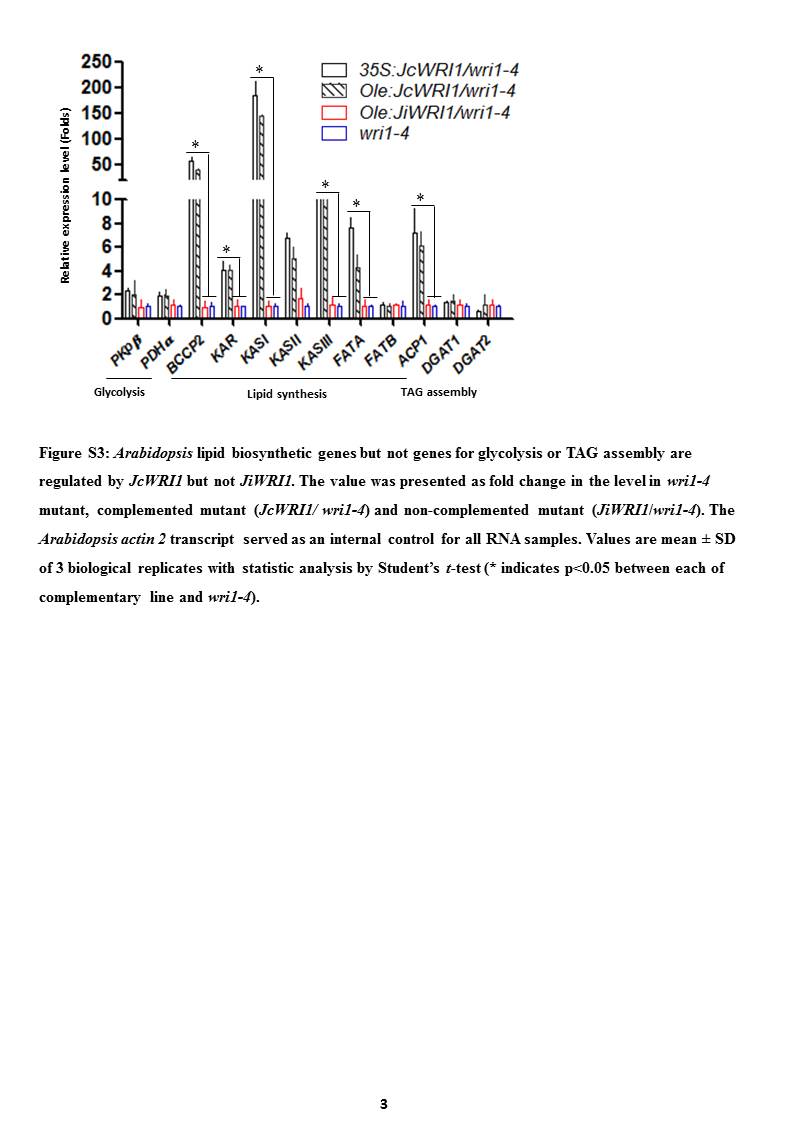

Supplement: Supplementary file 3 [file Image_3.JPEG]

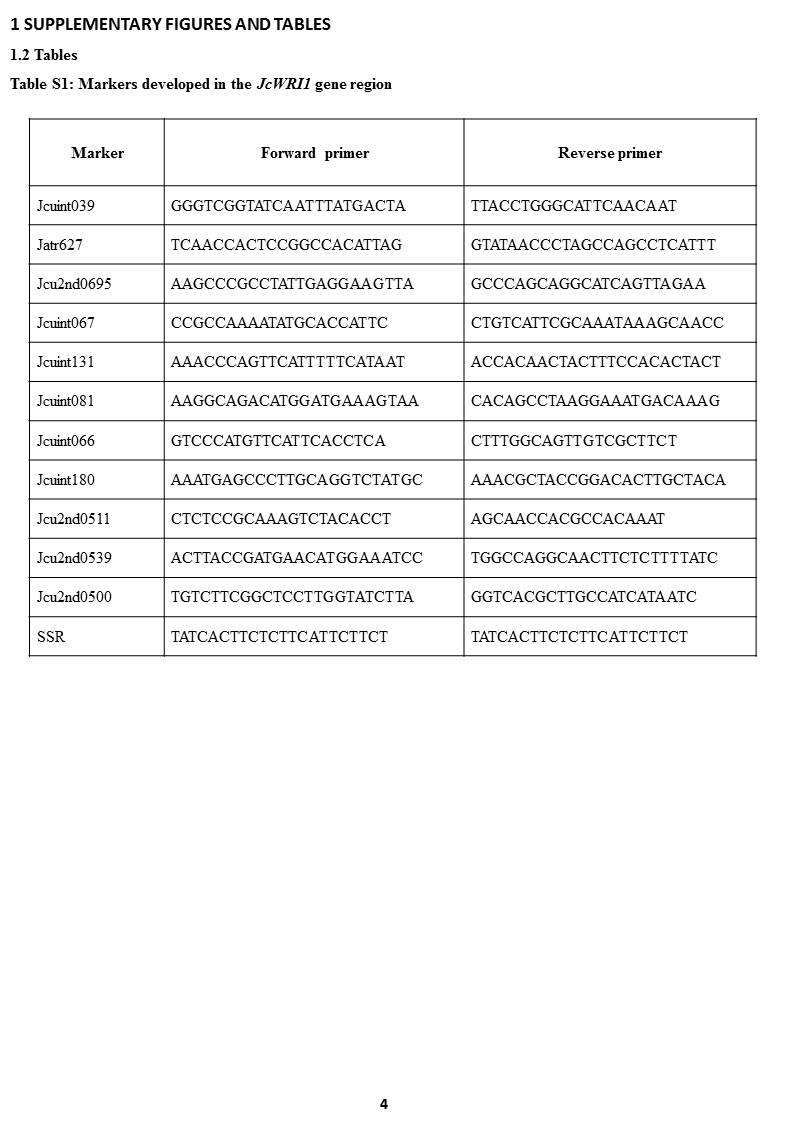

Supplement: Supplementary file 4 [file Image_4.JPEG]

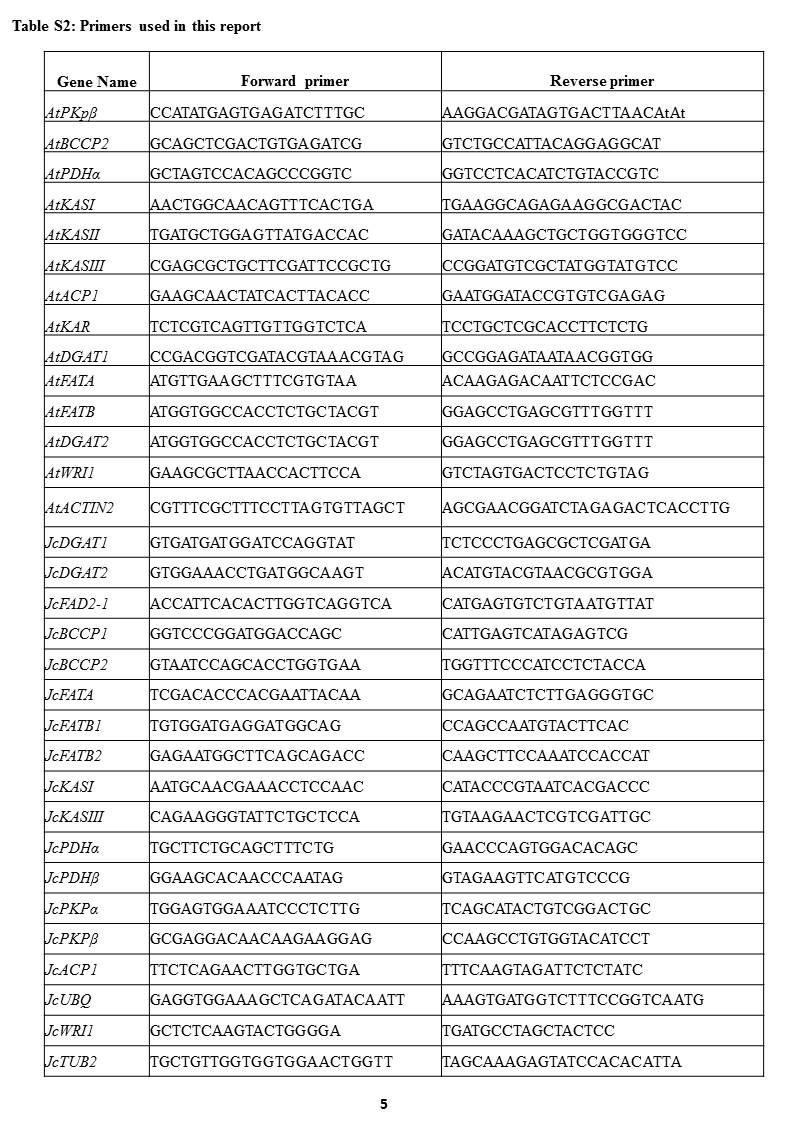

Supplement: Supplementary file 5 [file Image_5.JPEG]
